# Practice Patterns in the Treatment and Monitoring of Acute T Cell–Mediated Kidney Graft Rejection in Canada

**DOI:** 10.1177/2054358117753616

**Published:** 2018-02-15

**Authors:** Julie Leblanc, Peter Subrt, Michèle Paré, David Hartell, Lynne Sénécal, Tom Blydt-Hansen, Héloïse Cardinal

**Affiliations:** 1 Division of Internal Medicine, Department of Medicine, Université de Montréal, Québec, Canada; 2 Canadian National Transplant Research Program, Montreal, Québec, Canada; 3 Institut de recherche en santé publique de l’Université de Montréal, Québec, Canada; 4 Division of Nephrology, Department of Medicine, Hôpital Maisonneuve-Rosemont, Montreal, Québec, Canada; 5 Division of Pediatric Nephrology, University of British Columbia, Vancouver, Canada; 6 Division of Nephrology, Centre hospitalier de l’Université de Montréal, Québec, Canada; 7 Centre de recherche du Centre hospitalier de l’Université de Montréal, Montreal, Québec, Canada

**Keywords:** acute cellular rejection, protocol biopsy, clinical practices

## Abstract

**Background:**

One of the goals of the Canadian National Transplant Research Program (CNTRP) is to develop novel therapies for acute rejection that could positively affect graft outcomes with greater efficacy or less toxicity. To develop innovative management strategies for kidney graft rejection, new modalities need to be compared with current clinical practices. However, there are no standardized practices concerning the management of acute T cell–mediated rejection (TCMR).

**Objectives:**

To describe clinicians’ practice patterns in the diagnosis, treatment, and monitoring of acute TCMR in Canada.

**Design:**

Survey.

**Setting, Patients/Participants:**

Canadian transplant nephrologists and transplant surgeons involved in the management of acute TCMR.

**Methods and Measurements:**

We developed an anonymous, web-based survey consisting of questions related to the diagnosis, treatment, and monitoring of TCMR. The survey was disseminated on 3 occasions between June and October 2016 through the Canadian Society of Transplantation (CST) kidney group electronic mailing list.

**Results:**

Forty-seven respondents, mostly transplant nephrologists (97%), originating from at least 18 of the 25 Canadian centers offering adult or pediatric kidney transplantation, participated in the study. Surveillance biopsies were used by 28% of respondents to screen for kidney graft rejection. High-dose steroids were used by most of the respondents to treat clinical and subclinical Banff grade 1A and 1B rejections. Nine percent (95% confidence interval [CI]: 1-17) of practitioners used lymphocyte-depleting agents as the first-line approach for the treatment of Banff grade 1B acute rejection. Eighteen percent (95% CI: 7-29) and 36% (95% CI: 8-65) of respondents reported that they would not use high-dose steroids for treating clinical and subclinical borderline rejections, respectively. Seventy percent (95% CI: 54-83) of respondents answered that there was no indication to assess histological response to treatment independent of the change in kidney function.

**Limitations:**

The limitations of this study are its limited sample size and the low representation of pediatric specialists.

**Conclusions:**

There is heterogeneity regarding the use of surveillance biopsies, treatment of borderline rejection, and modalities to monitor treatment response among transplant physicians. Our results illustrate the current state of practice patterns across Canada and can be used to inform the design of future trials.

## Abrégé

**Contexte:**

Un des objectifs du Programme national de recherche en transplantation rénale du Canada (PNRTC) est de développer des traitements plus efficaces et moins toxiques en vue d’améliorer l’issue des greffes. Il est impératif de comparer ces nouvelles modalités aux pratiques cliniques existantes si l’on veut élaborer des stratégies de prise en charge thérapeutiques innovantes. Cependant, en contexte de greffe rénale, il n’existe aucune pratique standardisée pour la prise en charge thérapeutique du rejet aigu à médiation cellulaire (RAMC) provoqué par la cytotoxicité des lymphocytes T.

**Objectif:**

Décrire le schéma de pratique des médecins canadiens en matière de diagnostic, de traitement et de monitorage du RAMC.

**Type d’étude:**

Il s’agit d’une étude menée sous forme de sondage.

**Participants:**

Les chirurgiens et néphrologues en transplantologie impliqués dans la prise en charge du RAMC au Canada.

**Méthodologie:**

Nous avons préparé un sondage Web anonyme constitué de questions relatives au diagnostic et au monitorage du RAMC. Les répondants visés étaient les abonnés à la liste d’envoi du groupe de transplantation rénale de la Société canadienne de transplantation (SCT). Ils ont reçu le sondage à trois reprises entre juin et octobre 2016.

**Résultats:**

Les répondants, au nombre de 47, étaient en grande majorité des néphrologues transplantologues (97 %). Ils provenaient d’au moins 18 des 25 centres hospitaliers canadiens dans lesquels on pratique des greffes rénales (adultes ou pédiatriques). Vingt-huit pour cent (28 %) des répondants ont recours aux biopsies de surveillance pour évaluer le risque de rejet du greffon. Les stéroïdes administrés à fortes doses sont employés par la plupart des répondants pour traiter les rejets cliniques et infracliniques de stade 1A et 1B (classification de Banff). Les agents de déplétion des lymphocytes sont utilisés par 9 % (IC 95 % : 1-17) des praticiens comme approche thérapeutique de première ligne pour les rejets aigus de stade 1B de Banff. En matière de traitement des cas rejets limites cliniques et infracliniques, 18% (IC 95 % : 7-29) et 36 % (IC 95 % : 8-65) des répondants ont indiqués qu’ils n’emploieraient pas de stéroïdes à forte dose. Enfin, 70 % (IC 95 % : 54-83) des spécialistes sondés jugeaient qu’il n’y avait pas d’indication d’évaluer la réponse histologique au traitement indépendamment de la réponse au traitement en terme de fonction rénale.

**Limites de l’étude:**

Les résultats du sondage sont limités par le faible nombre de répondants et par la sous-représentation des spécialistes en pédiatrie.

**Conclusion:**

Chez les médecins sondés, on a constaté des différences dans trois aspects de la prise en charge de la greffe rénale : la fréquence du recours aux biopsies de surveillance, le traitement des cas limites de rejet et les modalités employées pour mesurer la réponse au traitement. Nos résultats témoignent de l’hétérogénéité actuelle des schémas de pratique au Canada et pourraient servir à orienter la conception d’études ultérieures.

## What was known before

To our knowledge, this is the first published survey that evaluates Canadian practice patterns regarding diagnosis, treatment, and monitoring of acute T cell–mediated rejection (TCMR).

## What this adds

This article describes current clinical practices related to acute TCMR across Canada and provides information in terms of design, feasibility, and acceptability of future research protocols for TCMR.

## Background

Acute T cell–mediated rejection (TCMR) occurs in slightly over 15% of kidney transplant recipients in the first 5 years after transplantation.^1^ Increased immunosuppression, including corticosteroid pulses, and the use of lymphocyte-depleting agents are the cornerstone of therapy, but their use entails significant toxicity such as infection and cancer.^2^ One of the goals of the Canadian National Transplant Research Program (CNTRP) is to develop novel therapies for treatment of TCMR that could positively affect graft outcomes with greater efficacy or less toxicity. More specifically, as part of the CNTRP Project 4, we are planning to study photodynamic therapy (PDT) as a treatment for acute TCMR. In small studies and case reports, PDT has effectively treated or prevented TCMR in heart, lung, liver, and renal transplant patients.^3,4^

To evaluate the efficacy of PDT or other novel treatment modalities, the latter will need to be compared with current clinical practices. A major problem inhibiting reliable comparison of novel treatments in multisite clinical trials is the absence of a uniform standardized protocol to treat TCMR in Canada. Given the weak strength of the evidence on which clinical guidelines are based^2^ and the heterogeneity of existing literature,^5^ there is also no uniformly accepted clinical conduct related to the use of surveillance biopsies, the treatment of subclinical acute rejection, the definition of response to treatment, and how it should be monitored.^2,5,6^ Hence, the aim of this study was to gather clinicians’ views and practice patterns on the diagnosis, treatment, and monitoring of acute TCMR in Canada.

## Methods

### Design

We developed an anonymous, web-based survey with the aim of gathering information on the diagnosis, treatment, and monitoring of acute TCMR in Canada.

### Instrument

The survey was initially developed in English by 3 transplant nephrologists (T.B.H., L.S., and H.C.) in a paper format. The survey questions were translated to French by a bilingual research assistant and revised by one of the investigators (H.C.). Both French and English versions were then built into a web-based tool (P.S.) using the FluidSurvey online system licensed to the University of British Columbia. The survey consisted primarily of 34 multiple-choice questions (using skip logic in the web-based tool) divided in 6 categories: questions related to the qualification of survey participation, diagnosis and surveillance of acute TCMR, treatment of clinical acute TCMR, treatment of subclinical acute TCMR, monitoring of the response to treatment in cases of TCMR, and demographic information of the respondents (see Additional File 1). The web-based survey was beta-tested by 5 transplant nephrologists to ensure adequate clarity and flow of the tool. We estimated that the time required to fill the survey was approximately 10 minutes. The study was approved by the Centre hospitalier de l’Université de Montréal’s ethics committee (reference number: 16.034). The first page of the survey explicitly mentions that it is a research project funded by the CNTRP. Consent was presumed by participation.

### Procedure

The link to the survey was transmitted to the Canadian Society of Transplantation (CST), who forwarded it to the members of the kidney group through a blast email on June 2, 2016. The CST kidney group includes 196 professionals who have self-identified as being involved in the field of kidney transplantation (transplant surgeons, nephrologists, nurses, and other allied health professionals). Contact was made with one transplant nephrologist in each Canadian transplant center to advertise the survey. A reminder containing the link was sent 1 month later by the CST. The investigators presented the study to the kidney transplant group at the annual CST meeting in October 2016, following which a last blast email was sent on October 20, 2016. The study closed on November 16, 2016. None of the participants received incentives. The participants’ responses were returned anonymously to the study team, although participants wanting to participate in further trials could leave their email address. The CST did not provide the research team with any information that could have led to identify the study participants.

### Inclusion and Exclusion Criteria

The survey targeted respondents who were involved in decisions related to the treatment of acute rejection. Hence, the first section of the survey comprised qualification questions. To ensure that respondents were involved in clinical work and making the decisions related to the surveillance and treatment of TCMR, we excluded participants who devoted less than 11% of their professional time per week to clinical work (cutoff point chosen by consensus), who were not actively involved in diagnosing and treating acute kidney graft rejection or did not answer the qualification questions.

### Data Analysis

The data were entered into IBM SPSS Statistics, version 23. Categorical variables are reported as proportions and continuous variables as means and standard deviations or median and interquartile ranges depending on their distribution. To account for the uncertainty around the estimates provided, we report 95% confidence intervals (CIs) for means and proportions. We compared therapeutic choices by rejection grade and center volume (dichotomized at the median value of 90 transplantations per year) using chi-square or Fisher exact tests.

## Results

**Figure 1.**
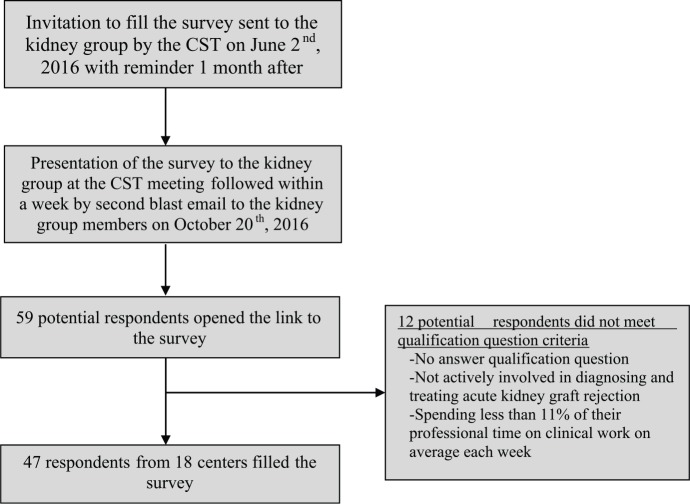
Flowchart of survey procedures and respondents. *Note.* CST = Canadian Society of Transplantation.

Among the 196 health professionals who were members of the CST kidney group, 59 potential respondents opened the link to the survey. Of those, 7 did not answer the qualification questions, 1 was not actively involved in diagnosing and treating acute kidney graft rejection, and 4 were not spending sufficient professional time on clinical work. The remaining 47 respondents, mostly transplant nephrologists (97%), affiliated with 18 of the 25 Canadian transplant centers were eligible for inclusion (Figure 1). Most respondents (n = 43, 91%) practice in adults transplant centers. Thirty-two percent of respondents (n = 12) had 11 to 20 years of experience, and 39% (n = 15) had more than 20 years of experience. Respondents were spending on average 63% of their professional time on clinical work each week (Table 1). Mean survey completion time was 8 minutes 43 seconds (interquartile range: 5 minutes 50 seconds to 12 minutes 30 seconds). On average, transplant centers were performing 89 ± 53 kidney transplantations per year and 37 ± 15% of kidney transplantations originated from living donors (Table 2).

**Table 1.** Characteristics of the Respondents (n = 47).

Specialization (%)	
Nephrologist	46 (97)
Transplant surgeon	1 (3)
Population treated (%)	
Adult	43 (91)
Pediatric	4 (9)
Percent time spent on clinical duties per week, mean (SD)	63 (22)
Years in practice, number of respondents, n = 38 (%)	
Still in fellowship training	1 (3)
Less than 5 years	2 (5)
5-10 years	8 (21)
11-20 years	12 (32)
More than 20 years	15 (39)
Working at the institution where they trained, n = 38 (%)	
Yes	20 (53)
No	18 (47)
Language in which the survey was completed, n = 46 (%)	
English	43 (91)
French	3 (9)
Province of origin, n = 38 (%)	
British Columbia	4 (11)
Alberta	3 (8)
Saskatchewan	1 (3)
Manitoba	1 (3)
Ontario	12 (32)
Quebec	11 (29)
Nova Scotia	2 (5)
Decline to answer	4 (11)

*Note.* Unless otherwise specified in the table, the number of respondents is not always equal to 47 as some questions were unanswered.

**Table 2.** Characteristics of Centers Represented (n = 18).

Person in charge of the long-term follow-up of kidney transplant patients at respondent’s center (%)	
Nephrologists	37 (97)
Both nephrologists and transplant surgeons	1 (3)
Patient population (%)	
Adult	43 (91)
Pediatric	4 (9)
Mean number of kidney transplants performed per year (SD)	89 (54)
Mean percent living (versus deceased) donor transplantations (SD)	37 (15)

### Monitoring and Diagnosing Acute Rejection

**Figure 2.**
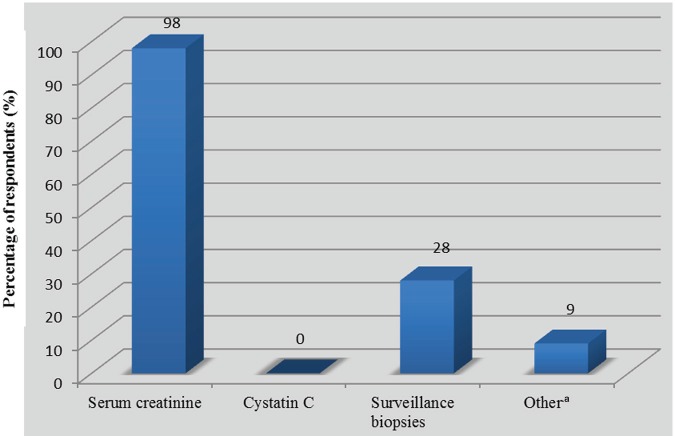
Tests routinely used to screen for the risk of kidney graft rejection (n = 46). ^a^Two for-cause biopsies, 1 albumin-to-creatinine ratio, and 1 uninterpretable.

The vast majority of respondents (n = 45, 98%, 95% CI: 94-100) routinely used serum creatinine to screen for kidney dysfunction as an indicator of rejection. The use of surveillance biopsies to screen for subclinical rejection was reported by 13 respondents (28%, 95% CI: 15-41). Cystatin C was not used to screen for rejection (Figure 2). On average, a 20 ± 8% increase in creatinine above baseline triggered a kidney biopsy to exclude rejection when other diagnoses had been excluded. Forty-one respondents (89%, 95% CI: 79-97) “always or almost always” awaited biopsy results before initiating treatment. Four participants (9%, 95% CI: 2-20) responded that they “usually” and 1 (2%, 95% CI: 0-12) that they “sometimes” waited for biopsy results before starting treatment. Among the 2 latter categories, respondents reported that they would promptly start treatment in the absence of biopsy confirmation if, on average, there was a 44 ± 34% increase in serum creatinine above baseline.

### Treatment of Clinical TCMR

#### First-line therapy

Therapies used for clinical TCMR are reported in Table 3. All respondents opted to treat clinical borderline rejection, although 1 respondent felt uncertain. To treat borderline rejection, 36 respondents (80%, 95% CI: 65-90) used high-dose steroids and 29 (67%, 95% CI: 51-80) increased maintenance immunosuppressants. Eight respondents (18%, 95% CI: 7-29) chose to optimize exposure to maintenance immunosuppressants without administering pulse corticosteroids, and 15 (33%, 95% CI: 20-47) said they would treat with high-dose intravenous (IV) or oral steroids alone without changes in maintenance immunosuppression. Twenty-one respondents (47%, 95% CI: 32-61) treated borderline rejection with a combination of high-dose steroids and optimized exposure to maintenance immunosuppressants. Banff grade 1A rejections were treated with high-dose corticosteroids by all 45 respondents (100%, 95% CI: 92-100). Of the latter, 30 (67%, 95% CI: 92-100) said they would also increase maintenance immunosuppressants, while 15 (33%, 95% CI: 19-47) would not. Banff grade 1B rejections were treated with high-dose corticosteroids by 44 respondents (98%, 95% CI: 88-100). Of the latter, 13 (29%, 95% CI: 16-42) administered steroids without changing maintenance immunosuppression, 28 (62%, 95% CI: 48-76) said they would also increase maintenance immunosuppression, and 3 (7%, 95% CI: 0-14) would administer lymphocyte-depleting agents in addition to steroids and increased maintenance immunosuppression. One respondent (2%, 95% CI: 0-7) said he would use lymphocyte-depleting agents as first-line therapy and only modality for grade 1B TCMR. Reported use of high-dose steroids was similar for Banff grade 1A and Banff grade 1B and significantly higher compared with borderline rejection (*P* < .01 for both comparisons). Increased exposure to maintenance immunosuppressants and lymphocyte-depleting agents was not statistically different according to the rejection grade. There was no difference in the use of steroids or in increased exposure to maintenance immunosuppressive agents according to center volume for each rejection grade. The 4 respondents who used lymphocyte-depleting agents as first-line therapy for grade 1B rejections came from 4 different centers.

**Table 3.** Clinical TCMR, First-Line Therapy Used by Banff Class (n = 45).

Therapies alone or combined	Borderline		Grade 1A		Grade 1B	
	n	% (95% CI)	n	% (95% CI)	n	% (95% CI)
Increased exposure to maintenance immunosuppressants,^a^ no steroid	8	18 (7-29)	0	0 (0-0)	0	0 (0-0)
IV methylprednisolone/PO prednisone alone	15	33 (20-47)	15	33 (20-47)	13	29 (16-42)
IV methylprednisolone/PO prednisone AND increased exposure to maintenance immunosuppressants^a^	21	47 (32-61)	30	67 (53-80)	28	62 (48-76)
Lymphocyte-depleting agents, IV methylprednisolone/PO prednisone AND increased exposure to maintenance immunosuppressants^a^	0	0 (0-0)	0	0 (0-0)	3	7 (0-14)
Lymphocyte-depleting agents, alone	0	0 (0-0)	0	0 (0-0)	1	2 (0-7)
Other^b^	1	2 (0-7)	0	0 (0-0)	0	0 (0-0)

*Note.* TCMR = T cell–mediated rejection; CI = confidence interval; IV = intravenous; PO = oral.

a Increase the dose and target through level of primary immunosuppressant (eg, calcineurin-inhibitors or mammalian target of rapamycin inhibitors) and/or optimize the dose of the adjuvant immunosuppressant (eg, mycophenolate mofetil or azathioprine) to the maximum tolerated.

b Uncertain: would give some IV steroid if persistently elevated creatinine, especially if the reason for TCMR is low tacrolimus level or reduced mycophenolic acid. If not, might elect not to treat, watch closely and consider repeating biopsy.

#### Corticosteroid doses and protocols

Most respondents (n = 34, 83%, 95% CI: 71-94) preferred pulsed IV methylprednisolone over high-dose oral prednisone. The most commonly reported protocols for IV methylprednisolone were almost evenly split between daily doses of 250 mg or 500 mg given for 3 consecutive days (highest dose reported; Table 4). After high-dose steroid treatment, IV or not, 34 study participants (87%, 95% CI: 77-98) reported tapering corticosteroids back to the baseline dose. While close to 50% reported a short taper of 7 to 14 days, 13 respondents (38%, 95% CI: 22-55) reported tapering over a period of 1 month and over. All pediatric specialists preferred methylprednisolone IV for the treatment of rejection.

**Table 4.** Corticosteroid Protocols for the First-Line Treatment of Clinical Rejection in Adult Centers (n = 41).

Protocols	n	% (95% CI)
IV methylprednisolone	34	83 (71-94)
Methylprednisolone 250 mg IV daily for 3 days, n = 30	14	40 (23-59)
Methylprednisolone 500 mg IV daily for 3 days, n = 30	10	33 (17-53)
PO prednisone	7	17 (6-29)
Corticosteroids tapering after high-dose course, n = 39	34	87 (77-98)
Duration 7-14 days, n = 34	16	47 (30-64)
Duration 1 month and over, n = 34	13	38 (22-55)

*Note.* The number of respondents is 41 unless otherwise specified. CI = confidence interval; IV = intravenous; PO = oral.

### Second-Line Therapy

We asked respondents to describe the circumstances that would prompt them to use second-line therapy with lymphocyte-depleting agents. Without performing a control biopsy, 12 of 42 respondents (29%, 95% CI: 15-42) said they would use lymphocyte-depleting agents if graft function was not improving, while 17 (40%, 95% CI: 26-55) would initiate such therapy if graft function deteriorated. When a control biopsy was performed, lymphocyte-depleting agents were reportedly used by 33 respondents (79%, 95% CI: 66-91) when the grade of rejection was similar and by 36 (86%, 95% CI: 75-96) when the grade was worse. Persistence of rejection, although of lower grade, would prompt the use of lymphocyte-depleting agents in 11 respondents (26%, 95% CI: 13-40), which was significantly lower than the proportion reported for similar or worse grade rejections (*P* < .01).

### Treatment of Subclinical TCMR

Data on the treatments used for subclinical TCMR are reported in Table 5. To treat subclinical borderline rejection, 7 respondents (64%, 95% CI: 31-89) used high-dose steroids and 9 (82%, 95% CI: 48-98) increased maintenance immunosuppressants. A single therapeutic approach was used by 36% of respondents, with 3 (27%, 95% CI: 6-61) choosing to optimize exposure to maintenance immunosuppressants alone without administering pulse corticosteroids, and one (9%, 95% CI: 0-41) using high-dose IV or oral steroids alone. Six respondents (55%, 95% CI: 23-83) used a combined approach with high-dose steroids and optimized exposure to maintenance immunosuppressants. Subclinical Banff grade 1A rejections were treated with high-dose corticosteroids by 10 respondents (91%, 95% CI: 59-100), and 11 (100%, 95% CI: 71-100) participants increased maintenance immunosuppressants. All physicians who chose to give high-dose steroids also increased maintenance immunosuppressants, while 1 (9%, 95% CI: 0-41) only did the latter. Subclinical Banff grade 1B rejections were treated exactly as subclinical Banff grade 1A. Reported use of high-dose steroids was not statistically different between rejection grades (*P* = .17 for the comparison between Banff grade 1A and Banff grade 1B vs borderline rejection). Increased exposure to maintenance immunosuppressants was not statistically different according to the rejection grade (*P* = .47). Responses for questions related to subclinical rejection episodes originated from 6 different centers, while 2 participants preferred not to identify their place of practice. Hence, the responses came from a minimum of 6 to a maximum of 8 different centers. Three respondents were from the same center, while all other centers were represented by 1 respondent.

**Table 5.** Subclinical TCMR, First-Line Therapy Used by Banff Class (n = 11).

Therapies alone or combined	Borderline			Grade 1A-1B		
	n	%	(95% CI)	n	%	(95% CI)
None	1	9	(0-41)	0	0	(0-29)
Increased exposure to maintenance immunosuppressants^a^, no steroids	3	27	(6-61)	1	9	(0-41)
IV methylprednisolone/PO prednisone alone	1	9	(0-41)	0	0	(0-29)
IV methylprednisolone/PO prednisone AND increased exposure to maintenance immunosuppressants^a^	6	55	(23-83)	10	91	(59-100)

*Note.* TCMR = T cell–mediated rejection; CI = confidence interval; IV = intravenous; PO = oral.

a Increase the dose and target through level of primary immunosuppressant (eg, calcineurin-inhibitors or mammalian target of rapamycin inhibitors) and/or optimize the dose of the adjuvant immunosuppressant (eg, mycophenolate mofetil or azathioprine) to the maximum tolerated.

### Monitoring for Response to Treatment of Acute Rejection

**Figure 3.**
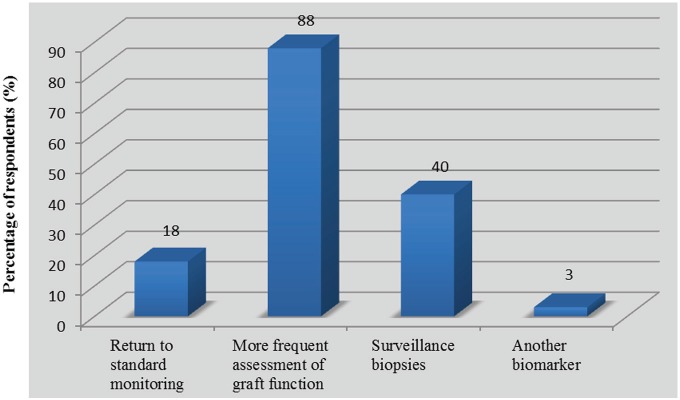
Monitoring strategies used after the treatment of kidney graft rejection (n = 40).

**Figure 4.**
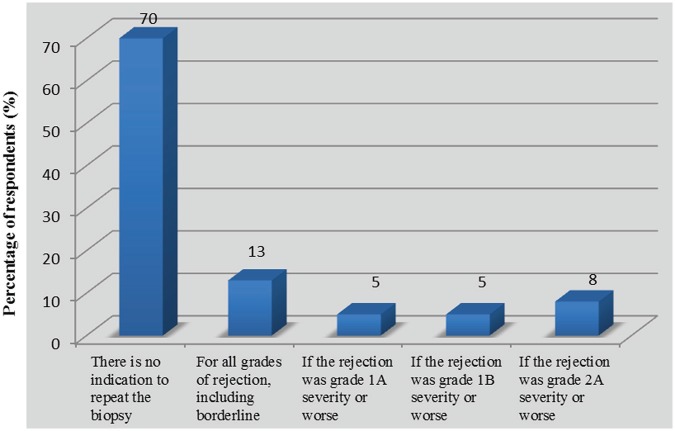
Percentage of respondents assessing histological response to treatment regardless of changes in graft function (n = 40).

**Figure 5.**
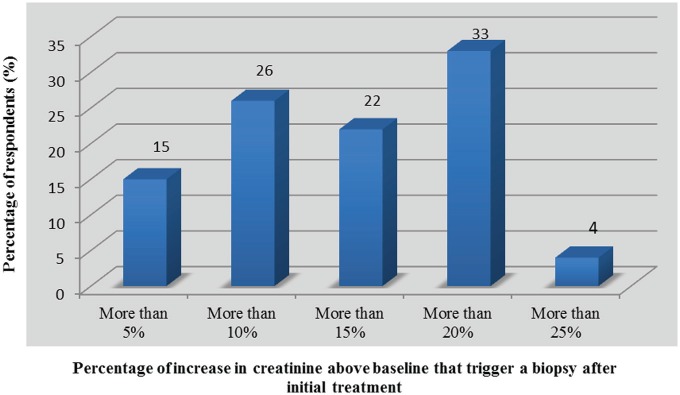
Changes in serum creatinine (in comparison with prerejection values) triggering request for a control biopsy (n = 27).

Following the initial treatment of acute rejection, 7 of 40 respondents (18%, 95% CI: 6-29) returned to standard monitoring of serum creatinine, while 35 (88%, 95% CI: 77-98) assessed kidney function more frequently and 16 (40%, 95% CI: 25-55%) reportedly used a control biopsy to assess histological clearance (Figure 3). Reversal of serum creatinine to prerejection values was a determinant factor in the decision to order a control biopsy, as 28 respondents (70%, 95% CI: 56-84) answered that there was no indication to assess histological response to treatment independent of the change in kidney function (Figure 4). This is consistent with the level of trust that physicians expressed with regard to changes in serum creatinine as a marker for the reversal of rejection (Table 6). Indeed, 31 respondents (78%, 95% CI: 65-90) felt “confident” or “somewhat confident” that rejection had resolved if creatinine was back to baseline levels. For respondents who based their decision to perform control biopsies on reversal of graft dysfunction, there was no consistent threshold to dictate when to perform a repeat biopsy. However, the vast majority of respondents (n = 26 of 27, 96%, 95% CI: 89-100) would not tolerate increases greater than 20% in serum creatinine without ordering a control biopsy (Figure 5). Twelve respondents (30%, 95% CI: 16-44) did assess histological response regardless of graft function depending on the initial severity of rejection (Figure 4). Among those performing control biopsies, there was no consistent threshold of severity to indicate the biopsy. The average time to repeat a biopsy was 5 ± 2 weeks after treatment.

**Table 6.** Trust Level of Resolution of Acute Rejection Depending on the Kidney Function (n = 40).

Trust level	Number of respondents, % (95% CI)					
	Kidney function					
	At the same level as the day of the biopsy		Partly but not completely back to baseline		Back to baseline	
Very confident	2	5 (0-12)	0	0 (0-9)	11	28 (14-42)
Somewhat confident	1	3 (0-8)	7	18 (6-30)	20	50 (35-65)
Not sure	8	20 (8-32)	12	30 (16-44)	6	15 (4-26)
Somewhat doubtful	10	25 (12-38)	12	30 (16-44)	2	5 (0-12)
Not confident	19	48 (33-63)	9	23 (10-36)	1	3 (0-8)

*Note.* CI = confidence interval.

## Discussion and Conclusions

To the best of our knowledge, this is the first published survey that specifically evaluates the clinical practices regarding diagnosis, treatment, and monitoring of acute TCMR in Canada. Our main findings are that serum creatinine remains the only noninvasive biomarker that is consistently used to monitor for graft dysfunction as an indicator of TCMR, with an average threshold in serum creatinine of 20% is used to trigger a graft biopsy for confirmation before treatment. Surveillance biopsies are performed by a minority (28%) of respondents. Another important consistency emerging from this survey is that high-dose steroids were used uniformly as first-line therapy to treat Banff grade 1A and 1B rejections. However, the doses used were variable, as were the presence and the length of a tapering period after high-dose pulses. First-line use of lymphocyte-depleting agents for grade 1B rejections was reported by a minority (9%) of respondents. The treatment of borderline TCMR was heterogeneous, as 18% and 36% of respondents reported that they would not use high-dose steroids for treating clinical and subclinical borderline rejections, respectively. Finally, the majority of respondents (70%) relied on the degree of reversal in graft dysfunction to decide whether a control biopsy should be performed to assess the histological clearance of rejection.

Although serum creatinine is known to be relatively insensitive to small changes in kidney function, it was the only noninvasive biomarker in clinical use to screen for the risk of rejection. While Kidney Disease: Improving Global Outcomes (KDIGO) guidelines state that researchers often arbitrarily use a 25% to 50% increase in serum creatinine above baseline to request biopsy,^2^ the average increase reported by our respondents was somewhat lower at 20%. No respondent used cystatin C, which does not seem to provide advantages over creatinine for predicting mild histological allograft changes.^7^ The identification of noninvasive biomarkers of inflammation and rejection is a very active area of research. Although not yet translated to the clinic, recent data suggest that urinary metabolomics and cytokine can play a role in noninvasive screening.^8-10^ In our study, 28% of practitioners used surveillance biopsies to screen for kidney graft rejection. In a recent United Network of Organ Sharing survey, 17% of US transplant centers performed protocol biopsies routinely on high- or low-risk patients (unpublished observation).^11^ The rationale for using surveillance biopsies is to provide an early opportunity to identify histological changes that cannot be predicted by biochemical measurements, leading to earlier intervention and, potentially, to improved graft outcomes. In contrast to their earlier work, Rush et al^12^ highlighted the low prevalence of subclinical rejection in the setting of current immunosuppression with tacrolimus, mycophenolate mofetil, and glucocorticoids. This recent trial failed to show histological or biochemical benefits in screening for and treating subclinical rejections.^12^ Although the safety of surveillance biopsies is well established,^13,14^ their risk-benefit and cost-benefit still need to be established. While their use remains a topic of controversy, they seem to be more likely to benefit populations at increased immunological risk, including those with a preformed donor-specific antibody or receiving blood ABO-incompatible transplants, pediatric patients, and those on calcineurin inhibitor or corticosteroids sparing regimens.^15-17^

While high-dose corticosteroids were uniformly used as the first-line treatment of clinical Banff grade 1A and 1B rejections, we found heterogeneity in the protocols used and the presence and length of a steroid-tapering period after high-dose treatment. Despite this heterogeneity, the reported doses, duration, and mode of administration were consistent with current guidelines.^2^ The optimization of maintenance immunosuppressants as a treatment modality was also variable. Some of the respondents increased the dose of the primary immunosuppressant, others optimized the adjuvant to the maximum dose tolerated, and others did both or none of these (see Additional File 2). We hypothesize that some of this variability is explained by the lack of clear evidence on these issues^2^ and by the fact that our survey could not capture subtleties in clinical reasoning, for instance, varying conduct related to the severity of rejection. Lymphocyte-depleting agents were generally regarded as second-line agents, although 9% of respondents used lymphocyte-depleting agents as the first-line approach in grade 1B rejections. This was not explained by clustering by center as the 4 respondents who elected to use this therapeutic approach originated from 4 different centers.

While most respondents treated clinical borderline rejection like grade I rejections, a significant minority (18%) would rather only increase the intensity of maintenance immunosuppressants and not give high-dose steroids as first-line therapy. In older studies, an eventual progression to rejection was documented in 30% to 50% of untreated patients with a rise in creatinine and borderline changes on the biopsy.^18,19^ Although there are no data on the evolution of such lesions in the era of modern immunosuppression,^20^ current guidelines suggest treating clinical borderline rejection. This recommendation is, however, of low grade (2D), and specific treatment protocols are not recommended.^2^

Although the treatment of subclinical rejection has demonstrated benefit almost exclusively in patients using a cyclosporine-based regimen^21,22^ and not in those with a tacrolimus-mycophenolate-based one,^12^ all respondents answered that they would treat grade 1A or 1B subclinical rejection. This is consistent with current guidelines, although again the recommendation is weak.^2^ One possible interpretation is that our survey, using skip logic, asked only the respondents performing surveillance biopsies to answer questions regarding the treatment of subclinical rejection. It is likely that their positive answer is consistent with their choice to perform the surveillance biopsies. These results may not be generalizable to clinicians who find subclinical rejection on biopsies requested for other motives than screening for rejection, such as delayed graft function, proteinuria, or hematuria. An area of even greater uncertainty is the treatment of borderline, subclinical rejection. The evolution of borderline lesions in untreated patients with stable graft function is controversial, and studies on the topic were performed in the era of less intense immunosuppressive protocols.^18,23,24^ There is no consensus on the treatment of subclinical borderline rejection, and Canadian Society of Transplantation and Canadian Society of Nephrology (CST/CSN) guidelines suggest treating subclinical borderline rejection based on clinical judgment, on a case-by-case basis.^17^ Treatment may be indicated for sensitized patients or those who already had a previous acute rejection episode.^17^ In this context, the heterogeneity in the responses we observed is expected.

We have identified another multicenter survey published in 1998 aimed to identify clinical practice and behaviors related to the diagnosis and management of acute kidney rejection and involved 17 directors of transplant programs internationally.^25^ Consistent with our results, this study showed that 77% of respondents defined successful response to treatment as a return to prerejection creatinine level. Older studies performed in patients with vascular (Banff 2-3) or corticosteroid-resistant Banff grade 1 rejections showed poor correlation between the reversal of serum creatinine and histological clearance of rejection on a posttreatment protocol biopsy.^26,27^ While maintenance immunosuppressive protocols have since changed,^20^ we have recently reviewed the literature^5^ and found no recent data reporting on the value of changes in serum creatinine to predict histological reversal.

The way to measure response to treatment in acute rejection is not addressed in KDIGO guidelines. A limitation of creatinine-based definitions of treatment response is their important variation across studies.^5,6,25,28^ Even though kidney function was back to baseline after treatment, 15% of our respondents were still unsure that acute rejection had been sufficiently treated, 5% were somewhat doubtful, and 3% were not confident. This may explain why 30% of respondents assessed histological response to treatment independent of changes in kidney function. While documenting the resolution of acute kidney rejection with a control biopsy is reassuring, the time frame needed for the resolution of the cellular infiltrate may vary and the significance of persisting inflammation on a biopsy in the face of complete biochemical recovery is unclear.^29^

Our study is informative for the conduct of future trials on the management of TCMR in various aspects. First, it provides information on what the standard of care is. In future studies, this can help investigators to design a control arm that is deemed acceptable by clinicians who would recruit patients. Second, it reveals areas where heterogeneity in practice patterns is greatest, for instance, the treatment of borderline rejection or performance of surveillance biopsies. As this heterogeneity is probably associated with lack of data, these areas represent research questions that need to be addressed. Finally, in areas of important homogeneity, such as in the use of steroids and/or increased maintenance immunosuppression in the treatment of grade 1A or 1B clinical rejection, it is important to note that future studies that do not contain an arm using this standard treatment may hardly be feasible given the high level of consensus.

Our study has various strengths and limitations. We succeeded in reaching respondents from at least 18 of the 25 Canadian kidney transplantation centers. We are unsure about the exact number of centers as our survey was anonymous. Respondents were asked to provide the name of their center, but this was not mandatory. Nevertheless, geographical representation was broad, as all provinces having a kidney transplant program were represented. Another limitation is that given the uncertainty around the center of origin, we did not adjust the confidence intervals around our estimates for potential clustering by center. As respondents from all provinces participated and given that only 1 center had 5 respondents, we are nevertheless confident that our results reflect practice patterns on a national basis and are not skewed by overrepresentation of 1 or 2 centers. After examining the CST membership list, we identified that among the 196 individuals who received an invitation to participate in our study, 53 were transplant nephrologists and 18 kidney transplant surgeons. The response rate was elevated among transplant nephrologists, given that 46 (87%) of those who received an invitation to participate did so. However, a limitation in terms of generalizability is that approximately half of transplant nephrologists in Canada are not CST members. After we analyzed our results, we made contact with all the 25 kidney transplant centers in Canada and identified 109 transplant nephrologists (24 pediatric, 85 adult). We identified 63 kidney transplant surgeons across Canada, although data for the latter could not be obtained for 1 adult and 2 pediatric centers. The response rate was low for transplant surgeons (5%), but as 97% of respondents answered that long-term follow-up and decisions on immunosuppression are made by nephrologists alone, we do not view this as a major limitation. Other limitations are that we obtained little representation of pediatric specialists and that even though we allowed respondents the opportunity to provide detailed responses to open-ended questions, many provided only a brief or no response. Finally, the small number of participants per center does not allow us to assess within-center variability in practice pattern and we could only analyze between-center variability by pooling centers according to volume, which showed no relationship with choice of therapy for clinical TCMR.

In conclusion, our study describes the current state of practice patterns in the diagnosis and treatment of acute tubulointerstitial rejection in Canada. Some aspects of management were uniform across all respondents. For instance, despite its lack of sensitivity, the only biomarker that is used clinically to screen for and evaluate the reversibility of rejection is serum creatinine. High-dose steroids were also used across the board as first-line treatment for clinical Banff grade 1A and 1B rejection. However, we also identified areas of heterogeneity, such as the treatment of clinical and subclinical borderline rejection and the decision to perform a control biopsy to assess the histological clearance of rejection. This heterogeneity speaks to the lack of data on these important issues, with most studies dating from era in which maintenance immunosuppressive protocols were less potent. Our results point to the need for more research in these areas of uncertainty and provide information for the design of future trials.
